# A Treatise on Diaphragmatic Hernia

**Published:** 1854-12

**Authors:** 


*A Treatise on Diaphragmatic Hernia*:—Being an account of a case observed at the Massachusetts General Hospital followed by a numerical analysis of all the cases of this affection found recorded in the writings of medical authors, between the years 1610 and 1846. By Henry G. Bowditch, M.D., one of the physicians to the Massachusetts General Hospital; member of the societies for medical observation of Paris and Boston. Buffalo, printed by Jewett, Thos & Co, 1853.

This is the title to a monograph of 77 pages, which first appeared in the Buffalo Medical and Surgical Journal. It is printed in good style and contains a detailed analysis of all that is at present known in reference to the rare and interesting form of hernia, of which it treats. The whole number of cases of diaphragmatic hernia which the author has been able to collect from the records of the last 250 years, is 88, one of which was observed by himself in the Massachusetts General Hospital.

This fact shows clearly enough, that hernial protusions through the diaphragm are of very rare occurrence. Of 68 cases, 26 were congenital, and 42 from accidental causes or injuries.

For obvious anatomical reasons, the hernia occurs in the *left* side of the diaphragm much more frequently than in the right.—Thus of 62 cases, 41 occurred on the left side, 18 in the right and three in both sides. Of 70 cases in which the sex is given, 53 were males and 17 females. Of the 26 congenital cases, 11 died within two hours after birth, 6 within two years, 1 at seven years, and 8 not until the adult age. Of 31 accidental cases death took place instantaneously in 4; within 24 hours in 12; within one week in 7; within one year in 5; two years and upwards 3 cases. One of the latter lived 38 years. The essay does our industrious friend, Dr. Bowditch, much credit; and we are sure it will be read with interest by all who seek an acquaintance with the rare things of our profession. D.

